# FT-IR and HPLC analysis of silver fir (*Abies alba* Mill.) bark compounds from different geographical provenances

**DOI:** 10.1016/j.heliyon.2024.e26820

**Published:** 2024-02-27

**Authors:** Irina M. Morar, Razvan Stefan, Catalina Dan, Radu E. Sestras, Petru Truta, Mădălina Medeleanu, Florica Ranga, Paul Sestras, Alina M. Truta, Adriana F. Sestras

**Affiliations:** a Department of Forestry, University of Agricultural Sciences and Veterinary Medicine, 400372, Cluj-Napoca, Romania; b Preclinic Department, University of Agricultural Sciences and Veterinary Medicine, 400372, Cluj-Napoca, Romania; c Department of Horticulture and Landscape, University of Agricultural Sciences and Veterinary Medicine, 400372, Cluj-Napoca, Romania; d Department of Food Science and Technology, University of Agricultural Sciences and Veterinary Medicine, 400372, Cluj-Napoca, Romania; e Faculty of Civil Engineering, Technical University of Cluj-Napoca, 400020, Cluj-Napoca, Romania; f Academy of Romanian Scientists, Ilfov 3, 050044, Bucharest, Romania

**Keywords:** *Abies alba*, Bark, Chemical compounds, Forest, FT-IR, Trees

## Abstract

Fourier Transform Infrared Spectroscopy (FT-IR) and High-Performance Liquid Chromatography (HPLC) could be applied to study the provenance of wood, specifically the differentiation of wood resources, as well as the identification of chemical compounds that are connected to the changes that occur in wood as a result of drying treatments. To test this hypothesis, the bark of silver fir (*Abies alba* Mill.) from trees belonging to seven different geographical provenances was studied, using samples dried at three different temperatures (60, 80, and 100 °C). FT-IR spectroscopy revealed different band assignments in the mid-infrared region depending on fir provenances, whereas the vibrational bands of the biomass functional groups tended to shift to lower wavenumbers. Significant differences were identified between the chemical compounds in the bark depending on the provenances. The largest proportion of the total phenolics was represented by the epicatechin gallate, epicatechin, catechin, and procyanidin dimer B1. Exploratory data analysis was performed using principal component analysis (PCA), hierarchical clustering, and Pearson correlations. This allowed a comparative evaluation of the samples and interpret the findings according to the geographical provenances, respectively ecological conditions in the areas of origin, but also the influence of the drying temperatures of the samples on chemical compounds. The precipitation in the areas of origin decreased total phenolics in silver fir bark samples, and total phenolics differed not only due to the geographic provenance, but also due to drying temperature.

## 1 Introduction

The life and biodiversity of the planet depend critically on forests, which also give people the necessary environmental, social, and economic circumstances for a proper life. Along with their numerous ecosystem functions and services [[1], [2], [3], [4]], forests also supply raw materials and wood for a variety of industries [1,5,6]. Wood is a complex lignocellulosic material composed of cellulose, hemicellulose, lignin, and various extractives. It is accepted that the wood of gymnosperms is of soft essence, and that of angiosperms is of hard essence, and they represent the main types of timber [7,8]. Wood is one of the most popular building materials, as well as being used for furniture and numerous other purposes [[9], [10], [11], [12]].

In the wood business, bark and branches are commonly considered waste; however, this could be a missed opportunity, because such materials can be valuable and are partially underutilized reserves [10]. The bark contains components similar to those present in wood, such as cellulose, hemicellulose, pectin, lignin and various extractive substances [13]. The bark and needles are used to extract the resin that is used for the production of turpentine, drugs, and cosmetics [14].

Information regarding variations in the proportions of the major organic components of wood can be obtained using the Fourier Transform Infrared Spectroscopy (FT-IR) method. It is a method that is frequently used to characterize wood and provides information on functional groups and molecular bonds [7,15], which is valuable for identifying wood components and determining the quality of wood [[16], [17], [18], [19]]. Furthermore, because extracts from the bark of diverse conifer species contain multiple types of polyphenols and have a diversity of actions, including pharmacological ones, reversed-phase High-Performance Liquid Chromatography (HPLC) has been commonly utilized to analyze the extracts of interest [[20], [21], [22]].

Analysis based on FT-IR spectroscopy in conjunction with multivariate statistical approaches provides numerous opportunities for study and data exploration; for example, FT-IR and principal component analysis (PCA) were effective in distinguishing between different wood species [[23], [24], [25]], to describe soil organic matter and microbial communities in forest sites [26], to identify the origin of lignin [27] and to detect pathogens, like *Fusarium* fungi [28]. FT-IR was developed as a tool for the simultaneous and quantitative determination of organic components (e.g., proteins, carbohydrates and lipids), including chemical bonds, being used in multiple fields [29]. PCA analysis has been also used to distinguish very subtle spectral changes in cell walls [30,31]. Gierlinger et al. [25] were able to differentiate between European larch (*Larix decidua*), Japanese larch (*L*. *kaempferi*) and hybrid larch (*L*. *eurolepis*) by means of FT-IR in combination with PCA, cluster analysis and independent class analogy modelling. Schimleck et al. [32] discriminated wood from different *Eucalyptus* species, from distant provenances, as well as from the same species grown in different locations, by analyzing NIR spectra by PCA.

Valid, rapid and simple methods of wood identification and certification are needed in forestry for several reasons, such as accurate recognition of the wood source, evaluation of different factors affecting the quality of wood, according to its destination, or even to prevent illegal logging and trade, by identifying and correlating specific components with the ecological conditions from where the trees were developing. Furthermore, forest microclimates influence biodiversity and ecosystem processes in forest landscapes [[33], [34], [35]], most likely also at the level of wood quality and critical wood components. Therefore, the main purpose of the current study was to validate a tool for evaluation of different provenances of Romanian silver fir (*Abies alba*), based on FT-IR and HPLC analyses of the silver fir bark, in order to identify genotypes with a high content of useful components, in regard with the quantity and quality of any lignocellulose or resin constituent and their use within industrial or medicinal purposes. The potential of FT-IR spectroscopy in combination with different multivariate statistical methods is lately used in forestry in order to distinguish wood of the same species, but harvested from different sources. Therefore, in the present study, silver fir bark samples collected from trees grown in seven different areas in Romania, were analyzed through FT-IR and cluster analysis, to identify their biochemical composition. Even more, it was aimed to have a more complete and comprehending image of the data regarding the silver fir bark, so that HPLC was pursued to gain more detailed results, thus to perform a more qualitative and quantitative analysis of the polyphenolic profile of bark extracts.

## 2 Materials and methods

### 2.1 Field sites and sampling

*A*. *alba* samples were collected from mature trees (between 60 and 80 years), chosen randomly to represent seven populations registered as seed source stands in Romania, located in different areas (Table 1).

**Table 1:** Administrative details of the studied *A*. *alba* provenances (populations) in regard with their provenance from Romania.

No.	Population	County	Administrative location	Latitude/Longitude
1	Valea Bistrei	Alba	O.S.P. Abrud, UP III, u.a. 228B	46°27′ N/23°01′ E
2	Someșul Rece	Cluj	O.S. Someșul Rece, UP I, u.a. 92A	46°38′ N/23°14′ E
3	Avrig	Sibiu	O.S. Izvorul Florii, UP III, u.a. 75A	45°37′ N/24°27′ E
4	Budescu	Maramureș	O.S. Poieni, UP IV, u.a. 96A	47°54′ N/24°36′ E
5	Sohodol	Alba	O.S.P. Abrud, UP IV, u.a. 18C	46°20′ N/23°06′ E
6	Valea Morii	Alba	O.S.P. Abrud, UP, u.a. 39	46°19′ N/22°56′ E
7	Gârda Seacă	Alba	O.S. Gârda, UP VI, u.a. 20H	46°31′ N/22°46′ E

**Fig. 1:**
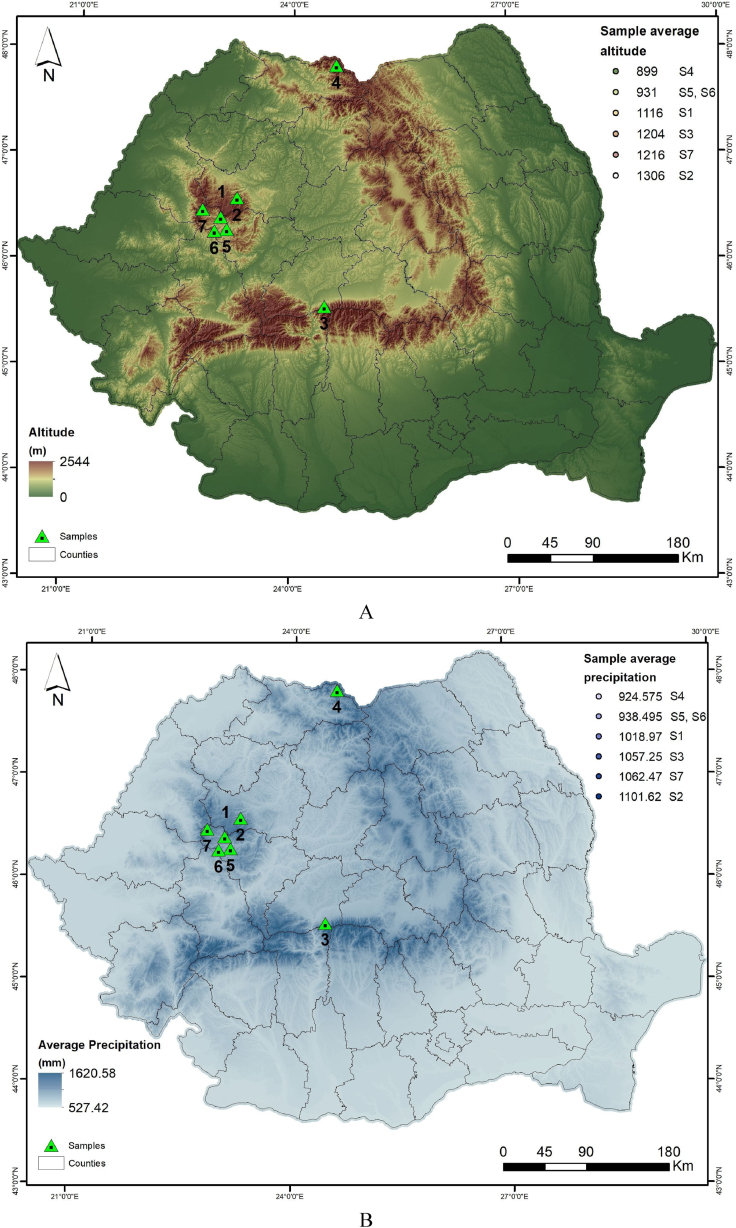

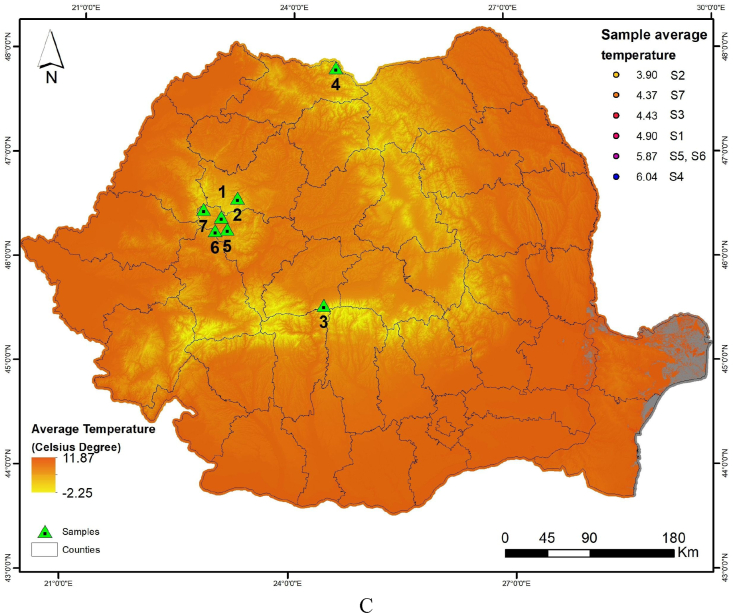
Location in Romania and the main parameters for investigated *A*. *alba* populations from which the bark samples were analyzed: Altitude, as m. a.s.l. (metres above sea level) (A); Amount of annual precipitation, as multiannual average, in mm (B); Average annual temperature, in °C (C). Data for mean annual temperature and mean annual precipitation are average values for a ten-year period (2012–2022) from meteorological stations.

The geographical origins of these provenances (P) were as follows: P1 – Valea Bistrei, P2 – Someșul Rece, P3 – Avrig, P4 – Budescu, P5 – Sohodol, P6 – Valea Morii, P7 – Gârda Seacă (Fig. 1A,B,C). Within all provenances, ten individual trees with similar growth characteristics (habitus and similar dimensions, a straight trunk, without defects, etc.) were randomly chosen for sampling. Stem bark samples were taken only from healthy trees, with no apparent damage. The samples have been taken at a trunk height of approximately 1.3 m above the ground, near the so-called ‘diameter at breast height’, or DBH), stored in paper tubes, and transported to the spectroscopy laboratory at the University of Agricultural Sciences and Veterinary Medicine of Cluj-Napoca. For FT-IR spectra were used ground bark, belonging to the repeated sampling of the same lot.

### 2.2 Analyses of silver fir bark by FT-IR

Circular incisions were made on the trunk of selected trees for each population, assuring that the proper bark for the investigation has at least 1 cm thickness. Before the FT-IR analysis, the stem bark samples were dried in the oven at different temperatures (60 °C, 80 °C and 100 °C) for a period of 2 h. The temperatures and the equal interval were established around those used in other similar studies [21,36,37]. In order to generate a composite sample for each provenance, bark samples have been chopped down into small pieces and then ground together in an equal contribution; Grinder Retsch Grindomix GM 200 was used at this stage, with mesh size <300 μm. After that, samples were made by combining 3 mg of the material with two hundred grams of calcined KBr [38]. In order to create a very fine powder, the sample and the KBr were both ground up together once again, until the mixture became completely homogenous. After placing the mixture in a steel spectral pellet kit, verification of the pellet was accomplished using a Specac hydraulic press.

In order to conduct the necessary measurements, each pellet was promptly inserted into a cassette of a Fourier Transform Infrared Spectometer model Jasco FT/IR 4100. The resolution was 4.0 cm^−1^, and the scanning range was between 4000 and 350 cm^−1^. Each spectrum was an average calculated from 256 scans that were carried out on a single sample pellet for each provenance. Spectra Manager was used to make five corrections for CO_2_ and five corrections for H_2_O for each spectrum. In order to perform a comprehensive study, peak reading was carried out using Origin by OriginLab [39].

### 2.3 Preparation of extracts

For the extraction of the phenolic compounds, 1 g of ground sample was weighted and extracted with 10 mL methanol, by vortex, for 1 min (Heidolph Reax Top Vortex). After this stage, the extract was placed in an ultrasonication bath for 1 h (Elmasonic E15H). During the final extraction step, the sample was centrifugated at 10,000 RPM, at room temperature, for 10 min (Eppendorf AG 5804). The collected supernatants were filtered through a micro-filter (Chromafil Xtra nylon 0.45 μm) and 20 μL were injected into the HPLC system.

#### 2.3.1 Phytochemical profiling – analysis of bark phenolic extracts by HPLC-DAD-ESI+

Regarding the chromatographic conditions, the separation and identification of the compounds were done by an HPLC system (HPLC Agilent 1200) equipped with a quaternary pump, solvent degasser, autosampler, a UV-VIS detector with a photodiode (DAD) and coupled with a single quadrupole mass spectrometer (Agilent Technologies 6110, CA, USA). The separation was performed on a Kinetex XB C18 column, 4.6 × 150 mm, 5 μm particle size (Phenomenex, USA), using water (A) and acetonitrile (B) as mobile phases, both containing 0.1% acetic acid (v/v), with a flow rate of 0.5 ml/min. The UV spectra were recorded in the 200–600 nm range for all the peaks, while the chromatograms were registered at the wavelength of 280 nm. The MS detector was used in ESI full positive ionization scanning mode: capillary voltage 3000 V, 350 °C, nitrogen flow 7 l/min and *m*/*z* 120–1200.

The data acquisition and interpretation of the results were performed using Agilent ChemStation software. Phenolic compounds have been identified by comparing retention time, UV-VIS and mass spectra with standard compounds and data from literature.

#### 2.3.2 Reagents and standards

Hydroxybenzoic acids were quantified as gallic acid equivalents and flavanols as catechin equivalents, so that we used the two phenolic compounds (gallic acid and catechin) as representatives of the polyphenols subclasses. Ultrapure water was purified using a direct-Q UV system from Millipore (USA), and HPLC-purity acetonitrile was acquired from Merck (Germany). Purity levels of >98% HPLC were achieved by using Sigma-Aldrich (USA) gallic acid and catechin.

Calibration curves were performed for the quantification of phenolic compounds by injecting different concentrations of standard substances dissolved in methanol, in the concentration range of 10–100 μg/ml for gallic acid and 10–200 μg/ml for catechin: for calibration curve of gallic acid, we used five concentrations: 100 μg/ml, 50 μg/ml, 25 μg/ml, 12.5 μg/ml, 10 μg/ml; for calibration curve of catechin the concentrations used were: 200 μg/ml, 100 μg/ml, 50 μg/ml, 25 μg/ml and 10 μg/ml respectively. The calibration curve of gallic acid has the equation y = 33.624x + 30.68 (R^2^ = 0.9978), LOD = 0.35 μg/ml, LOQ = 1.05 μg/ml; the calibration curve of catechin has the equation y = 15.224x – 130.24 (R^2^ = 0.9985), LOD = 0.18 μg/ml, LOQ = 0.72 μg/ml.

### 2.4 Data analyses

The final data were processed as mean total phenolic values. Analysis of variance (ANOVA) was utilized as a statistical test for a multifactorial experience to find possible differences between the means of the provenances and drying temperatures. Before applying the ANOVA test, the data were tested for normality. If the null hypothesis was rejected, ANOVA was completed using Duncan's test (α < 0.05) to separate and highlight the differences between means. Principal component analysis (PCA) and hierarchical clustering analyses utilizing the single linkage technique were performed using PAST software (PAleontological STatistics (PAST) Version 4.09, Natural History Museum, University of Oslo, Norway) [40]. The Pearson correlations between each phenolic compound, respectively total phenolics in the bark, and the main ecological conditions (i.e., temperature, precipitation, amplitude) were computed for the set of geographical provenances, and the graphical representations were made with the NCSS 2023 Statistical Software (NCSS, LLC, Kaysville, Utah) [41]. The P-values below 0.05 were considered statistically significant.

## 3 Results

### 3.1 The main FT-IR bands of the silver fir bark and their assignments

The FT-IR bands for the silver fir bark exposed to three different drying temperatures (respectively 60 °C, 80 °C and 100 °C), are presented in Table 2.

**Table 2:** Summary of peak positions for FT-IR spectra within 4000–350 cm^−1^ region of *A*. *alba* bark subjected to three different drying temperatures.

Peak no.	Wave number (cm^−1^)	Band origin	References
1	820–858	C–H out of plane in position 2, 5, and 6 of guaiacyl units	Faix, 1992 [42]; Boeriu et al., 2004 [43]
2	894	C–H deformation in cellulose	Pandey and Pitman, 2003 [44]
3	1029–1059	Aromatic C–H in plane deformation, guaiacyl type and C–O deformation, primary alcohol in cellulose	Hergert, 1971 [45]; Faix, 1991 [46]; Rana et al., 2008 [47]
4	1062–1076	C–O stretching of secondary alcohols	Faix, 1991 [46]
5	1103–1105	C–O–C stretching in cellulose and hemicellulose	McCann et al., 1992 [30]; Zhang et al., 2010 [48]
6	1146–1147	C–O–C asymmetric stretching in cellulose and hemicellulose	Faix and Bottcher, 1992 [42]; Popescu et al., 2007 [49]; Traoré et al., 2018 [7]
7	1206–1233	Syringyl (phenol) (S) nuclei deformation combined with deformation of cellulose	Evans et al., 1991 [50]
8	1267–1272	C–O vibration in guaiacyl rings	Popescu et al., 2007 [49]; Chen et al., 2010 [51]; Traoré et al., 2018 [7]
9	1315–1317	CH_2_ wagging in crystalline cellulose	Colom and Carrillo, 2005 [52]; Popescu et al., 2007 [49]; Traoré et al., 2018 [7]
10	1339–1374	C–H deformation in cellulose and hemicellulose	Pandey and Pitman, 2003 [44]; Evans et al., 1992 [53]; Mohebby, 2008 [54]
11	1451–1453	C–H deformation; asymmetric in –CH_3_ and –CH_2_- for lignins and hemicellulose	Hergert, 1971 [45]; Faix, 1991 [46]; Popescu et al., 2007 [49]; Chen et al., 2010 [51]; Traoré et al., 2018 [7]
12	1515	C=C stretching of the aromatic ring, C=O bond vibrations in extractive compounds	Popescu et al., 2007 [49]; Zhou et al., 2015 [18]; Traoré et al., 2018 [7]
13	1616–1618	C=O stretching conjugated to the aromatic ring, and in carboxylic groups in lignin, carboxylic acid, ester compounds	Zhao et al., 2014 [55]; Traoré et al., 2018 [7]
14	1620	Absorbed O–H and conjugated C–O in polysaccharides	Genest et al., 2013 [56]; Karunakaran et al., 2015 [57]; Traoré et al., 2018 [7]
15	1742–1746	C=O in ester groups, acetyl group in xylan, in unconjugated ketones, carbonyls and in ester groups (frequently of carbohydrate origin)	Bodirlau and Teaca, 2009 [17]; Zhou et al., 2015 [18]; Faix, 1991 [46]; Pandey and Pitman, 2003 [44]
16	2851–2860	CH_2_ stretching of cellulose and hemicelluloses	Longo et al., 2020 [58]
17	2923–2925	CH stretching of cellulose and hemicelluloses	Longo et al., 2020 [58]
18	3399–3426	H-bonded valence vibration, O–H valence vibration of C_(6)_H_2_O_(6)_–H primary alcohol (main conformation)	Fackler et al., 2010 [59]

One can assign the characteristic functional groups to a class of compounds. Thereafter, it can be recorded that bands in the range of 3399–3426 cm^−1^ correspond to compounds containing functional hydroxyl groups (-OH), including phenolic compounds (e.g., from lignin) such as gallic acids, protocatechuic acid, *p*-coumaric acid or aliphatic structures or hydroxyl group for alpha-cellulose.

Bands no. 13–15 match aldehyde groups (e.g., carbonyl C=O double bond stretching vibration in hemicelluloses (1850–1600 cm^−1^); the C=O stretching vibration of unconjugated carbonyl group in hemicelluloses (xyloglucan) was identified at 1742–1746 cm^−1^ (connected with the thermal treatment applied). The band no. 12, assigned to C=C vibration can be connected to aromatic ring vibrations of lignin. Further, C–O–C corresponds to vibrations of guaiacyl units, which are clearly evidenced within bark due to the high lignin content. The C–H in-plane bending vibration is represented by the interval 1500-900 cm^−1^. In lignin, cellulose, and hemicelluloses, C–O and C–C link to skeletal vibrations. Bands 5 and 6 correspond to cellulose and hemicellulose compounds with C–O–C stretching. Compounds with C–O stretching of secondary alcohols have bands in the 1062-1076 cm^−1^ range.

The principal alcohol C–O stretching vibration in cellulose was measured to be between 1029 and 1059 cm^−1^, depending on the heat treatment. The second peak at 894 cm^−1^ demonstrates aromatic C–H in-plane stretching, guaiacyl type, and C–O deformation. Guaiacyl-syringyl lignin (GS-lignin) found in hardwoods is a copolymer of coniferyl and sinapyl alcohols and was noted with different continents, starting from the C–H group present around 820-858 cm^−1^.

### 3.2 Assignment of FT-IR bands according to bark drying temperature

**Fig. 2:**
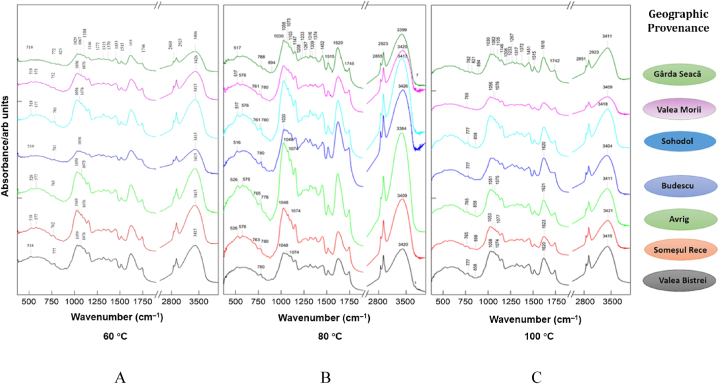
FT-IR band assignments in the mid-infrared region, after treating silver fir (*A*. *alba*) bark powder depending on the samples (seven geographical provenances) and the drying temperature: 60 °C (A); 80 °C (B); and 100 °C (C).

The results obtained using the FT-IR technique and three drying temperatures (60 °C, 80 °C and 100 °C) demonstrate variations in the chemical composition of the bark within the silver fir provenances (Fig. 2).

Silver fir bark showed 18 prominent peaks in the fingerprint region of the wavenumbers from 3500 to 800 cm^−1^ (Table 2), which represent major cell wall components (i.e., cellulose, hemicelluloses and lignin) [47]. The populations investigated registered comparable peaks, but some migration of bands was noted, depending on the provenance and the drying temperature (Fig. 2A,B,C). The migration of the CH_2_ stretching compound of cellulose and hemicelluloses from the wavenumber range of 2860–2851 cm^−1^, as well as the migration of the C=O stretch in unconjugated ketones, carbonyls, and ester groups, often originating from carbohydrates, from the wavenumber range of 1746–1742 cm^−1^, was observed in the provenance designated as Gârda Seacă.

FT-IR spectroscopy, which is often used to delineate the presence of the cellulose, hemicelluloses and lignin of different lingo-cellulosic materials [49], revealed different band assignments in the mid-infrared region depending on wood provenances. Strong absorption bands were observed in forest residues from fir bark attributed to O–H and C–H stretching vibrations (3500–3000 cm^−1^ and 3000–2650 cm^−1^), carbonyl C=O double bond vibration hemicelluloses (1850–1600 cm^−1^), C=C from aromatic skeletal vibrations, C–H plane bending vibrations and C–O and C–C skeletal vibrations (1500–900 cm^−1^) within lignin, cellulose and hemicellulose components. It can be noticed that the vibrational bands of the biomass functional groups had a tendency to shift (to lower wavenumbers), similar to other results [12,60]. Changes observed in the FT-IR spectrum for fir bark dried at 100 °C might be due to some different changes in the biomass compared to the samples treated at 60 and 80 °C, respectively changes attributed to unidentified compounds in this study. These bands became less sharp and wider with the increase in treatment temperature. The main bands associated with lignin components were detected between 1620 and 1616 cm^−1^ [7,55], while the cellulose and hemicelluloses were characterized by the bands between 2925 and 2923 cm^−1^ [58]. Group stretching O–H, as well as O–H in lignin, decrease significantly from 60 °C to 100 °C; for example, after the bark was exposed to 60 °C, the peak was found at 3426 cm^−1^ for Valea Morii provenance and migrated to 3409 cm^−1^ after 100 °C; similarly, for Budescu provenance, the band migrated from 3415 cm^−1^ to 3404 cm^−1^. For nearly all components, among the drying temperature tested for silver fir bark, the bands’ position migrated to lower wavenumbers from 60 °C to 100 °C. Such is the case of aromatic C–O vibration in guiacyl rings, which for the seventh provenance was located at 1272 at 60 °C treatment and shifted to 1267 at 80 °C and 100 °C.

Several studies present well-defined peaks which provide information on various functional groups present in fir wood constituents and their variation depending on the source (needles, bark) that can be identified [12,61]. Differences could also be noticed among samples, according to the wood provenances. This could be explained by the degradation of the lignin, which has become significant at high temperatures.

Peaks in the interval 3399-3426 cm^−1^ indicate an increase of free or only weakly H-bonded O–H groups of cellulose. These evolving O–H bands may suggest the depolymerisation of the polysaccharides and the relative resistance of cellulose crystallites.

### 3.3 Identification of phenolic compounds in silver fir bark samples by HPLC

**Fig. 3:**
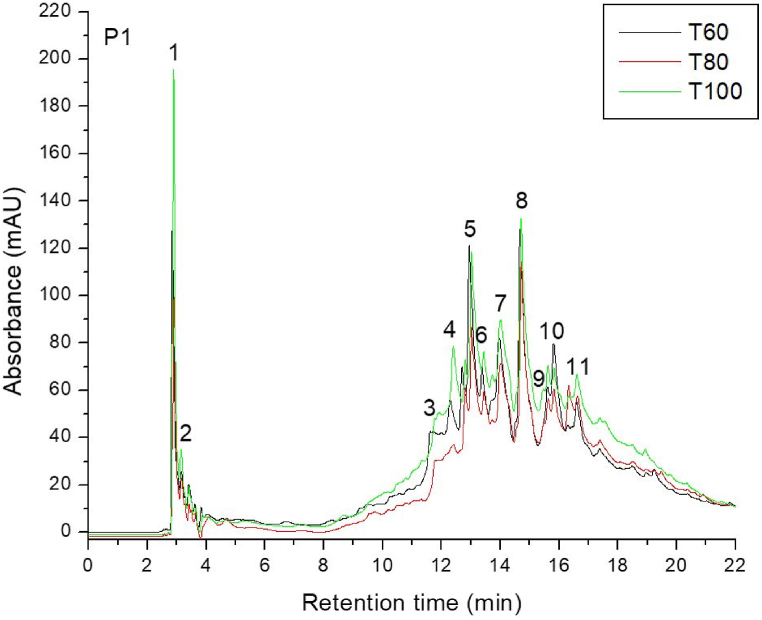
HPLC chromatogram for *A*. *alba* from P1 – Valea Bistrei provenance, for the three drying temperatures: T1 = 60 °C; T2 = 80 °C; T3 = 100 °C.

**Fig. 4:**
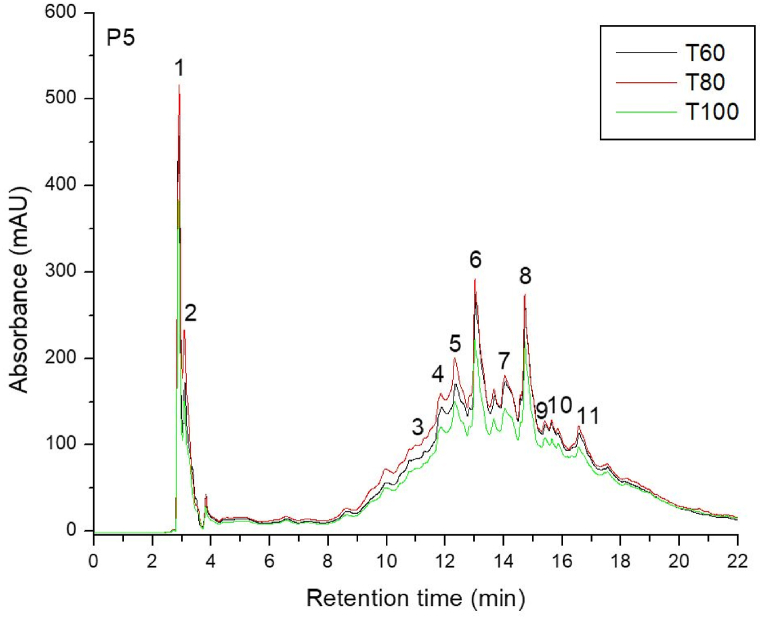
HPLC chromatogram for *A*. *alba* originated from P5 – Sohodol provenance, for the three temperatures: T1 = 60 °C; T2 = 80 °C; T3 = 100 °C.

Based on their MS fragmentation patterns, high-resolution mass, UV spectra, and retention times, 11 phenolic compounds in total were found in the 21 samples of powder silver fir bark. Representative chromatograms for two selective provenances, respectively P1 – Valea Bistrei and P5 – Sohodol, using the three drying temperatures: T1 = 60 °C; T2 = 80 °C; T3 = 100 °C are illustrated in Fig. 3, Fig. 4). These two provenances have approximately average values for the total phenolics among the populations analyzed, and thus can be considered convenient representations for all chromatograms obtained in the study. Flavonols were responsible for the greatest peaks in the HPLC traces of silver fir wood extracts, while the lower peaks were represented by hydroxybenzoic acids.

Peak number identities from all the analyzed provenances are displayed in Table 3, along with quantifications of phenolic compounds (mg/g) depending on the three drying temperature (T1 = 60 °C; T2 = 80 °C; T3 = 100 °C), in silver fir samples from seven geographic provenances, are presented in Table 4, Table 5, Table 6.

**Table 3:** Peak number identities and phenolic compounds identified in silver fir samples.

Peak No.	R_t_ (min)	UV *λ*_max_ (nm)	[M+H]^+^ (*m*/*z*)	Phenolic compound	Subclass
1	2.91	270	155	2,3-Dihydroxybenzoic acid	Hydroxybenzoic acid
2	3.16	270	139	2-Hydroxybenzoic acid	Hydroxybenzoic acid
3	11.59	280	307	Gallocatechin	Flavanol
4	11.96	280	579	Procyanidin dimer B3	Flavanol
5	12.42	280	291	Catechin	Flavanol
6	13.01	280	579	Procyanidin dimer B1	Flavanol
7	14.11	280	291	Epicatechin	Flavanol
8	14.78	280	443	Epicatechingallate	Flavanol
9	15.69	280	453	Catechin-glucose	Flavanol
10	15.89	290	153	Vanilin	Hydroxybenzoic acid
11	16.62	360, 260	303	Ellagic acid	Hydroxybenzoic acid

**Table 4:** Quantification of phenolic compounds (mg/g), at T = 60 °C (T1 – drying temperature), in silver fir samples from seven geographic provenances.

Peak No.	R_t_ (min)	Phenolic compound	Provenances						
			P1	P2	P3	P4	P5	P6	P7
			Valea Bistrei	Someșul Rece	Avrig	Budescu	Sohodol	Valea Morii	Gârda Seacă
1	2.91	2,3-Dihydroxybenzoic acid	0.182	1.105	1.343	0.067	1.055	0.160	1.051
2	3.16	2-Hydroxybenzoic acid	0.049	0.307	1.175	0.117	0.757	0.215	0.456
3	11.59	Gallocatechin	0.375	1.367	1.449	0.106	0.937	0.252	1.426
4	11.96	Procyanidin dimer B3	0.290	0.979	5.382	0.166	2.999	0.348	2.585
5	12.42	Catechin	0.796	2.354	5.641	0.201	3.862	0.566	4.414
6	13.01	Procyanidin dimer B1	0.772	2.149	5.388	0.217	4.577	0.602	3.664
7	14.11	Epicatechin	1.412	2.968	6.498	0.196	3.426	0.680	3.970
8	14.78	Epicatechingallate	1.441	3.356	5.252	0.239	4.250	0.811	3.568
9	15.69	Catechin-glucose	0.693	0.681	1.592	0.197	1.071	0.347	1.376
10	15.89	Vanilin	0.335	0.196	1.021	0.055	0.656	0.148	0.915
11	16.62	Ellagic acid	0.367	0.492	1.084	0.424	1.480	0.272	2.323
		Total Phenolics	6.713	15.954	35.825	1.985	25.070	4.400	25.747

**Table 5:** Quantification of phenolic compounds (mg/g), at T = 80 °C (T2 – drying temperature), in silver fir samples from seven geographic provenances.

Peak No.	R_t_ (min)	Phenolic compound	Provenances						
			P1	P2	P3	P4	P5	P6	P7
			Valea Bistrei	Someșul Rece	Avrig	Budescu	Sohodol	Valea Morii	Gârda Seacă
1	2.91	2,3-Dihydroxybenzoic acid	0.209	1.226	1.393	0.077	1.227	0.075	1.138
2	3.16	2-Hydroxybenzoic acid	0.050	0.594	1.167	0.089	1.060	0.123	0.946
3	11.59	Gallocatechin	0.334	1.666	1.667	0.096	1.022	0.109	2.379
4	11.96	Procyanidin dimer B3	0.281	2.114	5.365	0.135	3.541	0.202	3.306
5	12.42	Catechin	0.737	2.326	5.764	0.150	4.736	0.622	5.888
6	13.01	Procyanidin dimer B1	1.084	3.055	5.614	0.176	5.059	0.709	4.320
7	14.11	Epicatechin	1.399	4.449	5.432	0.123	3.728	0.543	5.451
8	14.78	Epicatechingallate	1.835	4.911	5.758	0.156	4.551	1.269	3.949
9	15.69	Catechin-glucose	0.470	0.771	1.799	0.168	1.140	0.156	2.150
10	15.89	Vanilin	0.342	1.000	0.986	0.025	0.766	0.168	1.324
11	16.62	Ellagic acid	0.434	1.116	1.130	0.925	1.736	0.322	2.630
		Total Phenolics	7.176	23.228	36.075	2.120	28.567	4.300	33.479

**Table 6:** Quantification of phenolic compounds (mg/g), at T = 100 °C (T3 – drying temperature), in silver fir samples from seven geographic provenances.

Peak No.	R_t_ (min)	Phenolic compound	Provenances						
			Valea Bistrei	Someșul Rece	Avrig	Budescu	Sohodol	Valea Morii	Gârda Seacă
1	2.91	2,3-Dihydroxybenzoic acid	0.191	0.648	0.750	0.096	0.844	0.266	0.694
2	3.16	2-Hydroxybenzoic acid	0.041	0.288	1.098	0.121	0.694	0.205	0.840
3	11.59	Gallocatechin	0.302	1.102	1.866	0.086	0.819	0.509	2.314
4	11.96	Procyanidin dimer B3	0.269	0.828	1.949	0.086	1.280	0.678	2.898
5	12.42	Catechin	0.656	1.933	4.876	0.086	3.275	0.218	5.595
6	13.01	Procyanidin dimer B1	0.722	1.764	5.593	0.086	3.512	0.200	4.290
7	14.11	Epicatechin	1.286	2.572	5.669	0.093	2.666	0.212	5.130
8	14.78	Epicatechingallate	1.738	3.125	6.366	0.086	3.328	0.772	4.054
9	15.69	Catechin-glucose	0.449	0.595	2.120	0.086	0.805	0.405	2.340
10	15.89	Vanilin	0.317	0.500	1.232	0.000	0.418	0.201	1.317
11	16.62	Ellagic acid	0.473	0.789	0.380	0.412	0.926	0.456	2.689
		Total Phenolics	6.445	14.144	31.899	1.237	18.566	4.122	32.159

The obtained chromatograms (21 in total, three temperatures for all seven provenances) look almost similar, whereas the amount of phenolic compounds differs depending on the area. It can be noted that the population with the highest content of phenolic compounds was represented by P3 – Avrig (35.825 mg/g at 60 °C (Table 4), 36.075 mg/g at 80 °C (Table 5), 31.899 mg/g at 100 °C (Table 6), while the one with the lowest content was P4 – Budescu (1.985 mg/g at 60 °C (Table 4), 2.120 mg/g at 80 °C (Table 5), 1.237 mg/g at 100 °C (Table 6). Also, the influence of temperature was marked and can be concluded that 80 °C seems to be the optimal temperature for polyphenol content analysis.

The most abundant compounds in the stem bark samples of silver pin were epicatechingallate, epicatechin, procyanidin dimer B1 and catechin, but their concentration was different depending on the drying temperature applied. Epicatechin and catechin are the first derivative products of flavan-3-ols, while gallocatechin forms polymeric tannins.

**Fig. 5:**
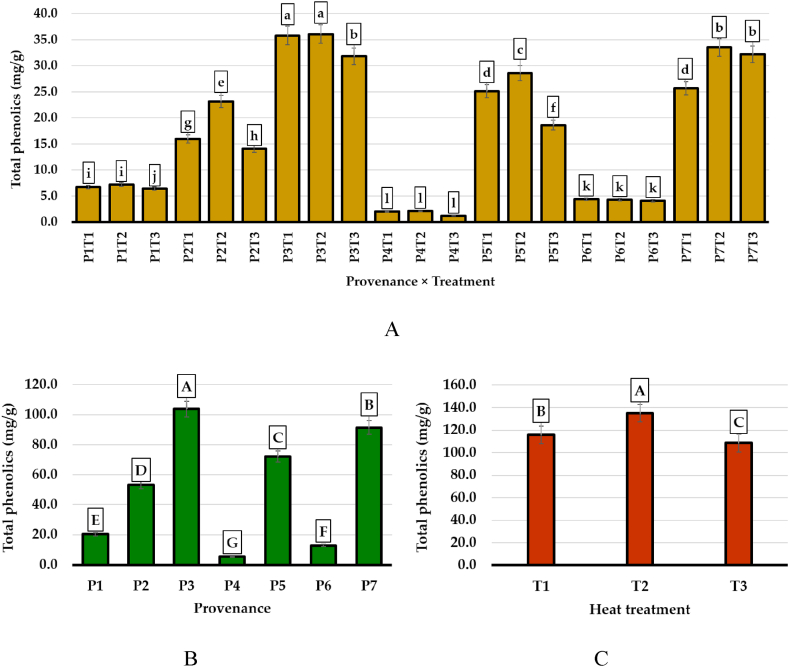
Total phenolic compounds (mg/g) identified in the bark belonging to different geographical provenances of silver fir, according to (A) the interaction between the geographical origins (seven provenances: P1 – Valea Bistrei, P2 – Someșul Rece, P3 – Avrig, P4 – Budescu, P5 – Sohodol, P6 – Valea Morii, P7 – Gîrda Seacă) and drying temperature (three levels: T1 = 60 °C; T2 = 80 °C; T3 = 100 °C); (B) the unilateral influence of geographical provenances, regardless of the drying temperature; (C) the unilateral influence of the drying temperature, regardless of the geographical provenance. Within the interaction between provenances and treatments (A), respectively, for geographical provenances (B) and treatments (C), significant differences between means are illustrated with different letters (Duncan's Multiple Range Test, p < 0.05).

The results presented in Fig. 5A highlight the significant differences between the content of the fir bark in total phenolics depending on the interaction between provenances and treatments. Drying temperature did not influence the variation of phenolics in the bark in only two provenances, P4 – Budescu and P6 – Valea Morii, which also had the lowest amount of total phenolics (Fig. 5B). Among the provenances, it stood out with a high content of total phenolics P3 – Avrig, followed by P7 – Gârda Seacă and P5 – Sohodol. Unlike the influence of the geographical origin of the samples, drying temperature had significant effects on the phenolics content (Fig. 5C). It had higher values at the treatment with 80 °C and lower at 100 °C, while the treatment with the lowest temperature provided intermediate values.

### 3.4 Multivariate analysis for A. alba provenances related to FT-IR and HPLC bark investigation

**Fig. A.1:**
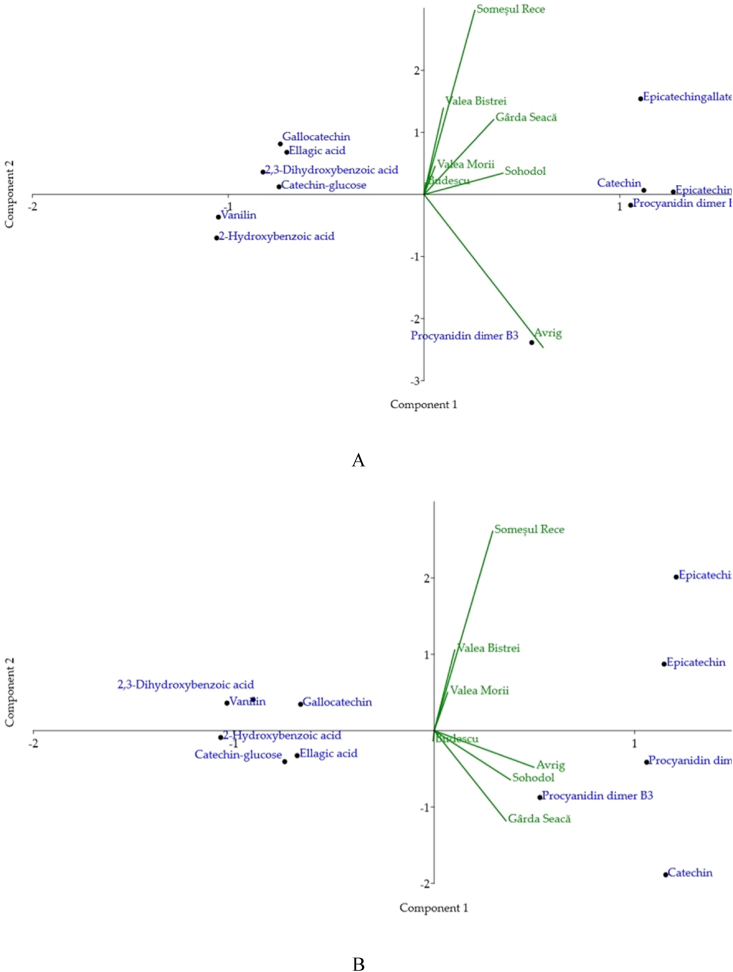

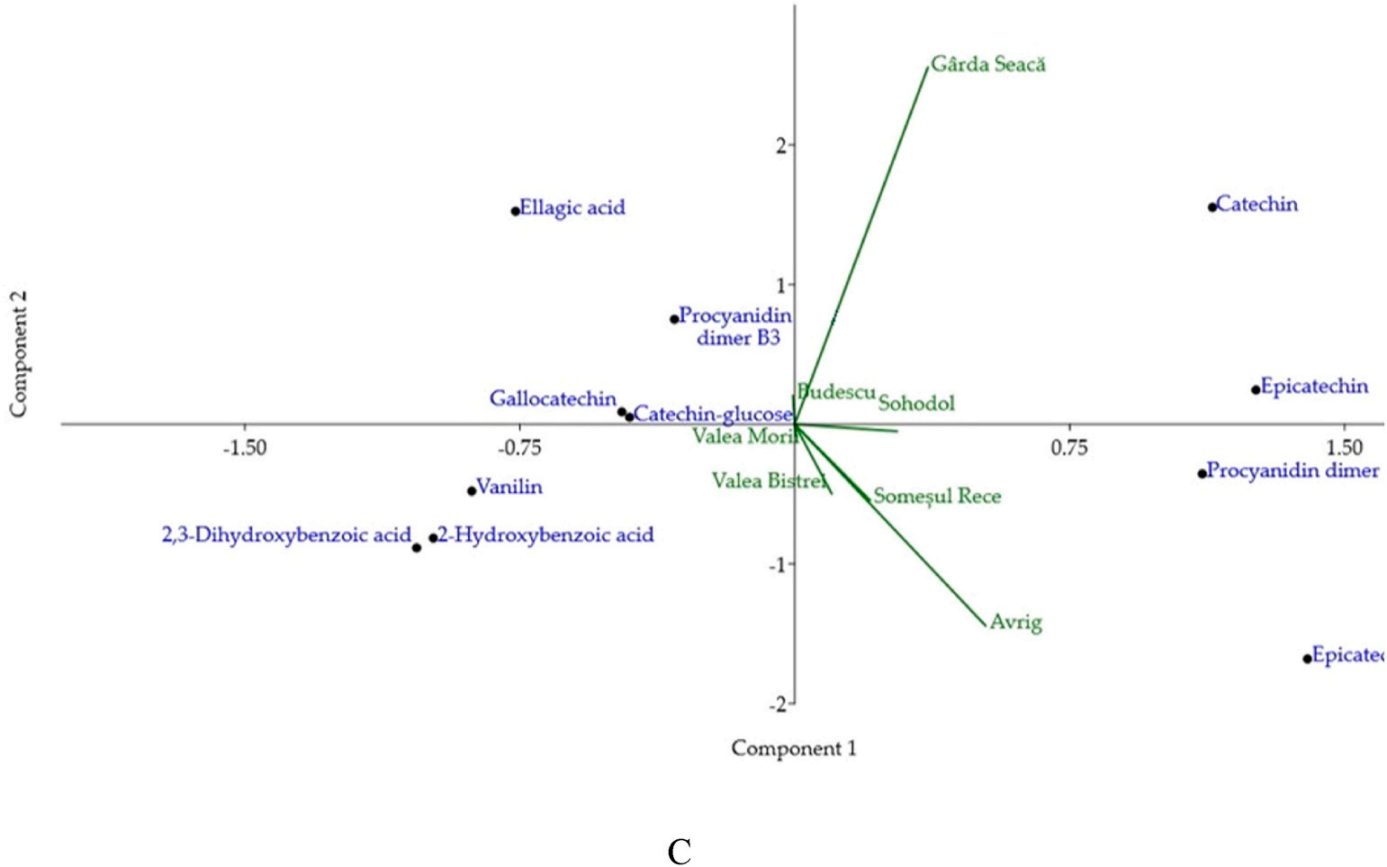
Summarizing datasets and information regarding the origins of silver fir (seven provenances) and phenolic compounds in the bark, using Principal Component Analysis (PCA), depending on the drying temperatures: (A) T = 60 °C (T1 – drying temperature); (B) T = 80 °C (T2 – drying temperature); (C) T = 100 °C (T3 – drying temperature).

Principal component analysis (PCA) was used to explore the three drying temperatures (T1 = 60 °C, T2 = 80 °C, and T3 = 100 °C) for *A. alba* bark, and the findings are displayed in Fig. A1 (A, B, and C). In the PCA dataset for the lowest drying temperature, T1 = 60 °C, component 1 explains 92.3%, while component 2 has 3.7% of the variation, so cumulatively, the two main components contribute 96.0% of the total variation. At T2 = 80 °C, component 1 accounts for 89.4% and component 2 has 5.4% of the total variation, whereas at T3 = 100 °C, component 1 explains 91.5% and component 2 accounted 5.3% of the total variation. Someșul Rece and Avrig appear as the most distant provenances. All the geographical origins are arranged in the quadrants on the right, quadrant I (upper right), and quadrant II (lower right), both at T1, and at the following two drying temperatures (T2 and T3). In all PCAs, the closest provenances were Valea Morii and Budescu. At T1 and T2, in opposite quadrants and at large distances, epicatechingallate and 2-hydroxybenzoic acid are located, and at T3 epicatechingallate and ellagic acid. At T3, on the other diagonal, catechin is found opposite and distant from 2,3-dihydroxybenzoic acid, 2-hydroxybenzoic acid, and vanillin.

**Fig. 6:**
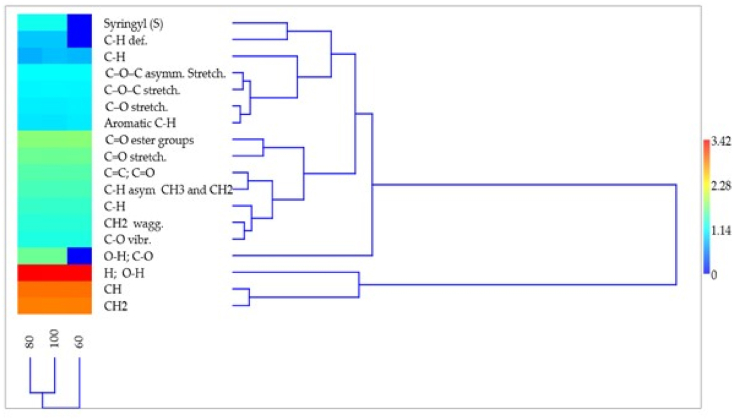
Hierarchical clustering – paired group UPGMA (unweighted pair group method with arithmetic mean) for 15 components identified in bark of seven provenances of *A*. *alba* using FT-IR technique, at three different drying temperatures (60, 80, 100 °C).

Hierarchical cluster analysis and the corresponding heatmap summarized the relationships between the chemical compounds, in the row dendrogram, but also between the three drying temperatures, in the column dendrogram (Fig. 6). The row dendrogram illustrates the clusters of compounds according to the grouping of observations that were made on the peaks and the levels of similarity between these. The cluster of phenolic compounds has two main branches, the lower one containing only three compounds, two of which are closely placed in a subcluster (CH and CH_2_) and one more distant (H, O–H). The upper subcluster has two branches, one represented by a single compound, but the other with numerous branches and subclusters, some finished as four pairs in which the compounds are very close (i.e., C–O–C asymm. Stretch. and C–O–C stretch.; C–O stretch. and Aromatic C–H; C=C, C=O and C–H *asym* CH_3_ and CH_2_; CH_2_ wagg. and C–O vibr.). The column dendrogram of drying temperatures shows a closer relationship between the 80 °C and 100 °C levels, which are grouped in a pair subcluster from which the 60 °C level appears more distant.

**Fig. A.2:**
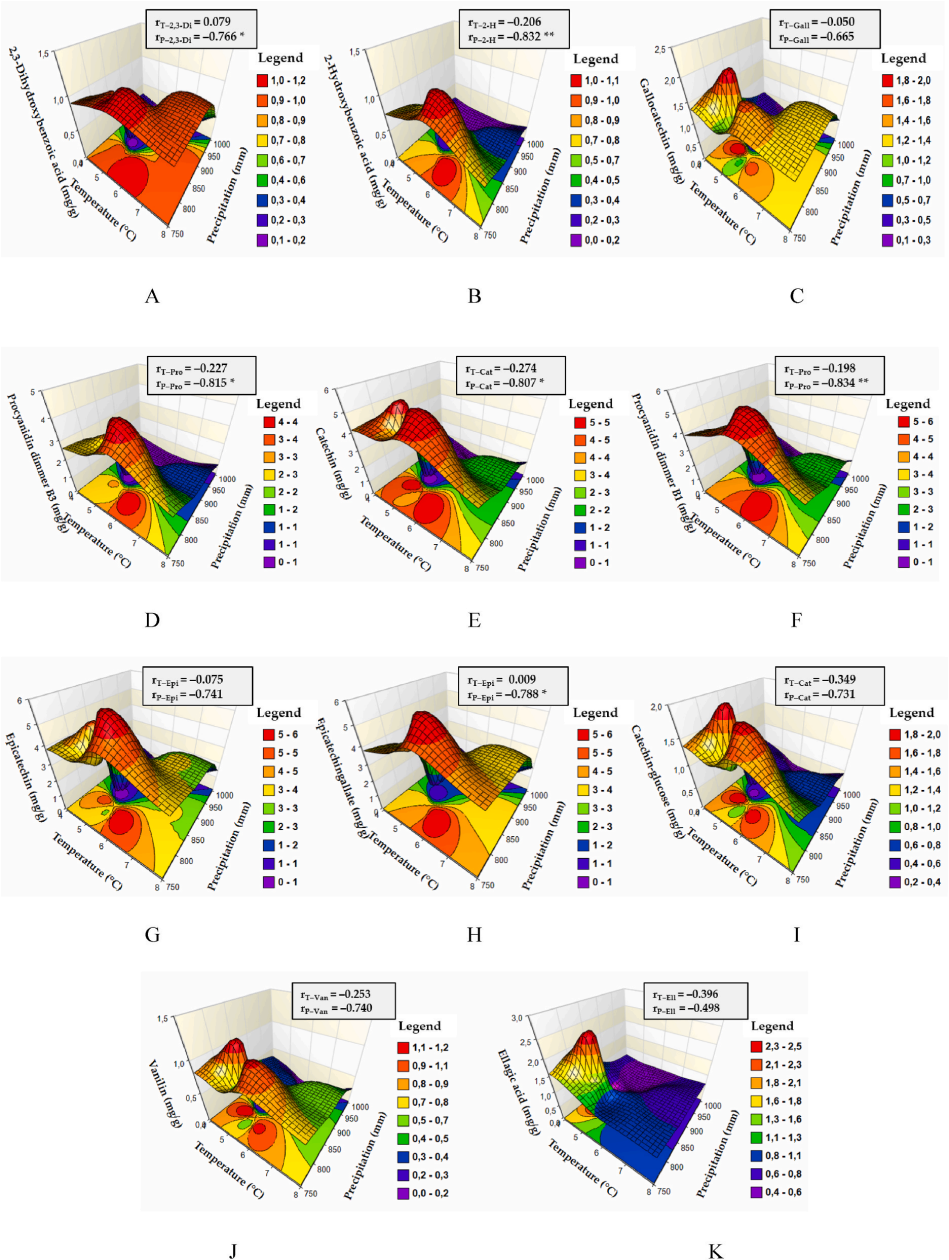
The Pearson correlations (‘r’) between the main ecological conditions (i.e., T–temperature, P–precipitation) of the silver fir geographic provenances, and phenolic compounds in the bark: (A) 2,3-Dihydroxybenzoic acid; (B) 2-Hydroxybenzoic acid; (C) Gallocatechin; (D) Procyanidin dimer B3; (E) Catechin; (F) Procyanidin dimer B1; (G) Epicatechin; (H) Epicatechingallate; (I) Catechin-glucose; (J) Vanilin; (K) Ellagic acid. The ‘r’ values considered statistically significant were symbolized by * (P value < 0.05) and ** (P value < 0.01).

**Fig. A.3:**
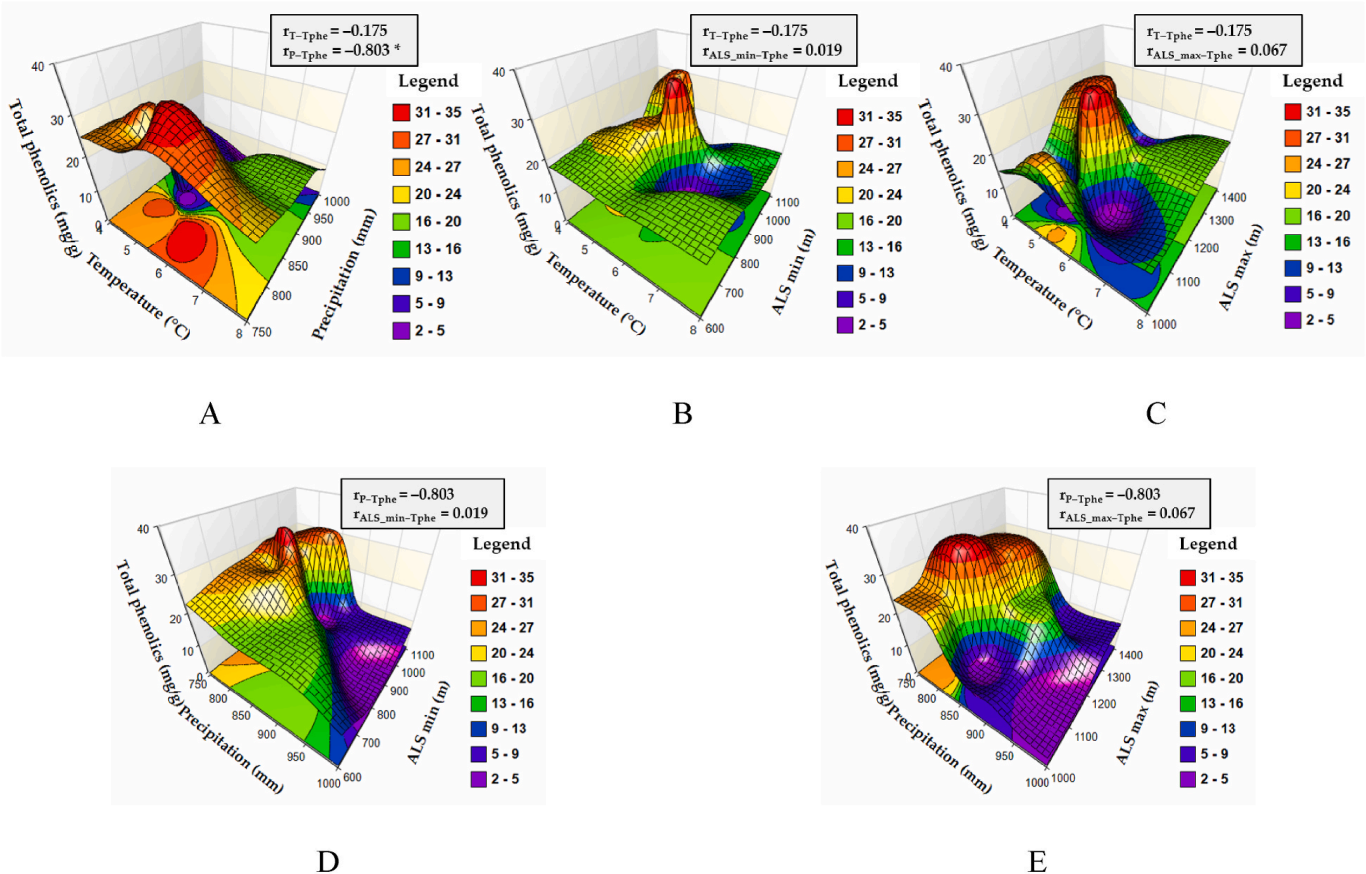
The Pearson correlations (‘r’) between the total phenolics in the bark of the silver fir provenances, and the main ecological conditions of the geographic provenances: (A) temperature, precipitation; (B) temperature, ALS min – minimum altitude; (C) temperature, ALS max – maximum altitude; (D) precipitation, ALS min – minimum altitude; (E) precipitation, ALS max – maximum altitude. The ‘r’ value considered statistically significant was symbolized by * (P value < 0.05).

The Pearson correlations calculated between the main ecological conditions (temperature, precipitation, altitude) of the silver fir geographic provenances and phenolic compounds in the bark revealed that the level of more than half of the phenolic compounds decreased significantly as precipitation increased (Fig. A2). A significant negative correlation (r = −0.803*) was identified both between the total phenolics and precipitation (Fig. A3), illustrating that the amounts of phenolic compounds in the bark are inversely associated with the quantity of precipitation in the areas of the silver fir geographic provenances.

## 4 Discussion

The current study is a new attempt to evaluate *A*. *alba* provenances using FT-IR fingerprints and HPLC. FT-IR approach is useful for analyzing wood because it provides information about functional groups and chemical bonds [15,49,50,62], lucrative for identifying wood parts, wood type, and also wood quality assessment [16,19].

FT-IR spectra have been effectively used to characterize the chemical part of wood and bark [42,44,46,62,63], as well as the detection and further the influence of fungi on wood [44,64]. Infrared spectroscopy has also been applied to distinguish tree species. Niemz et al. [65] were able to differentiate between softwoods and hardwoods based on chemical differences in the lignin composition of conifers and angiosperms which were easily detected by FT-IR analyses. However, if differences in chemical composition are small, analysis of individual chemical components is insufficient to group a set of samples by species [47,66].

Chromatography results further revealed that silver fir bark extracts contain large amounts of chemical compounds such as cellulose, hemicellulose, lignin, flavonoids, phenols with antioxidant activity and others, fact that corresponds to other studies carried out [67,68]. Within the same species, genetic variation in the forest can be manifested within and among populations [69]. It is of interest to uncover genetic differences that also determine phenotypic differences in trees, such as growth, resilience to stresses, and useable properties of wood [[70], [71], [72]].

Tree habitus, wood, bark, and chemical compounds can take different peculiarities within species and populations, and under specific ecological conditions, i.e. soil, climate, altitude, forest characteristics, etc. [73]. Bark thickness and constitution depend on the species, the age of the tree, the ecological factors from the origin area, and slightly on different parts of the tree [20]. Bark is also important because it guards trees’ stems from fire and other dangers and helps them absorb nutrients, connect to water, and often fix damage [74]. Bark, a non-technical name for tissues located outside the vascular cambium [75], that perform important physiological tasks in a living tree, includes the following: transport and storage of photosynthesis-derived compounds [76], as well as defence against biotic and abiotic stressors [77,78]. Its function can be deduced from the chemical composition, which varies according to the quantities and proportions of structural (cellulose, hemicelluloses, lignin, and suberin) and non-structural (extracts) substances [79]. Conifer bark can contain up to six times more extractives than stemwood (softwood) [78,79], and the bark comprises 10–15% of stem volume [20]. The bark of the silver fir tree comprises various extractable non-structural constituents, including soluble carbohydrates, terpenes, aliphatic alcohols, fatty acids, and polyphenols such as stilbens, flavonoids, lignans, and tannins [78]. The bark is comprised of various tissues encompassing the vascular cambium, encompassing both the periderm and secondary phloem, which is further divided into inner and outer bark [80]. The secondary phloem, which is the layer directly surrounding the vascular cambium, contains polyphenolic parenchyma cells, where the majority of the bark secondary metabolites are produced. These cells also include lipids, carbohydrates, and polyphenolic chemicals [81]. The utilization of bark from commercially cultivated softwood trees presents a promising avenue for accessing secondary metabolites, notably polyphenols such as tannins. These compounds have demonstrated utility in the production of adhesives and resins [20]. A higher total extractive content of the bark was identified in the upper positions of the silver fir trunk [20].

European softwood bark-derived condensed tannins are accepted as an alternative to synthetic phenolics [82]. Due to the composition of the majority of flavonols and acids, the tree bark represents an important source of materials for wood industry. Due to the hydroxyl groups’ reactivity with formaldehyde, condensed tannins are ideally suited for the efficient production of adhesives, polymers and foams. Our findings reveal that a significant quantity of catechins and epicatechins (a subgroup of flavanols) were detected in the bark of some provenances, confirming the necessity for proper utilization of this resource in silver fir [11,12,78]. Epicatechin and catechin have been identified in various kinds of trees, including leaf extracts from several *Salix species*, but also in a range of fruits, beans, and chocolate [83]. In comparison to samples from the other provenances, Gârda Seacă contained larger concentrations of ellagic acid. This polyphenol is present in the wood and bark of several forest species, including *Quercus*, *Eucalyptus*, and *Castanea* [84] and has been recognized having antioxidant, anticancer, and antimutagenic activities. As a result, waste from the forestry, wood-processing, and agro-forestry industries might be used to recover some bioactive chemicals, including ellagic acid [85].

Most infrared bands cannot be traced to a single component due to the intricacy of wood. To improve FT-IR spectral analysis, multivariate statistical approaches can be applied [51]. Similar to the current investigation, principal component analysis (PCA) has been used to discover chemical differences between early-wood and late-wood [86], or to discriminate between trees growing in different places [47]. The hierarchical clustering method enabled the combination of multiple information gathered from detailed sample characterization into a global perspective of the system. Multivariate statistical analysis is frequently used to make clear distinctions based on band positions obtained in FT-IR [51].

In our investigation, infrared spectroscopy and HPLC, in combination with statistical interpretation of data, were valuable instruments to evaluate provenance and characterize the noted differences between the studied samples. The findings provide a potential starting point for selecting appropriate resources represented by natural forests, as well as optimal processes for the utilization of wood from renewable biomass in energy and commodities with added value. Because of the finite nature of fossil-based resources and the negative environmental impact of their use, harnessing renewable biomass is gaining popularity in sustainable forestry. However, for future studies, an in-depth method will be conceived for the assessment of factors (except geographical, respectively genetic, and ecological) that may influence the chemical compounds of interest in fir bark, including age, condition, size of trees, and different sampling heights, cardinal points, different years, but also different seasons.

## 5 Conclusions

In order to identify prospective uses as sources of significant chemicals, this study includes an in-depth investigation of silver fir bark from seven different geographic provenances in Romania. Among the assessed sources, several populations with high potential for biomass utilization were found, according to the final and compositional analysis. Interpopulation variations of Romanian silver fir caused by genetic and ecological variables appear to be sources of a variety of chemical compounds of interest in tree bark, namely considerable levels of phenolics and flavonoids. Depending on the origins of *A*. *alba*, the extractive fractions of some provenances contained a significant amount of beneficial compounds and potential antioxidant activity. A negative correlation was identified between total phenolics and precipitation, meaning that as precipitation, respectively the altitude levels increase, the amounts of phenolic compounds in silver fir bark decrease.

## Data availability statement

Data associated with the study has not been deposited into a publicly available repository and data will be made available on request.

## CRediT authorship contribution statement

**Irina M. Morar:** Writing – original draft, Project administration, Investigation, Funding acquisition, Conceptualization. **Razvan Stefan:** Writing – review & editing, Validation, Supervision, Resources, Methodology, Formal analysis, Conceptualization. **Catalina Dan:** Writing – original draft, Investigation, Data curation. **Radu E. Sestras:** Writing – review & editing, Formal analysis. **Petru Truta:** Resources, Investigation, Data curation. **Mădălina Medeleanu:** Visualization, Investigation, Formal analysis. **Florica Ranga:** Visualization, Investigation, Formal analysis. **Paul Sestras:** Validation, Supervision, Software, Methodology. **Alina M. Truta:** Resources, Project administration, Methodology, Data curation. **Adriana F. Sestras:** Writing – review & editing, Supervision, Software, Methodology, Formal analysis, Conceptualization.

## Declaration of competing interest

The authors declare the following financial interests/personal relationships which may be considered as potential competing interests:Irina M. Morar reports financial support and article publishing charges were provided by 10.13039/501100006595UEFISCDI, 10.13039/501100015622Ministry of Research and Innovation, the project number PN–III–P1-1.1-PD-2021-0651. Irina M. Morar reports article publishing charges was provided by University of Agricultural Sciences and Veterinary Medicine from Cluj-Napoca (USAMVCN).
